# Improvement of Fatty Acid Profile in Durum Wheat Breads Supplemented with *Portulaca oleracea* L. Quality Traits of Purslane-Fortified Bread

**DOI:** 10.3390/foods9060764

**Published:** 2020-06-10

**Authors:** Maria Grazia Melilli, Vita Di Stefano, Fabiola Sciacca, Antonella Pagliaro, Rosaria Bognanni, Salvatore Scandurra, Nino Virzì, Carla Gentile, Massimo Palumbo

**Affiliations:** 1 Institute for Agricultural and Forest Systems in the Mediterranean, National Council of Research, 95128 Catania, Italy; antonella.pagliaro@isafom.cnr.it (A.P.); rosaria.bognanni@cnr.it (R.B.); salvatore.scandurra@cnr.it (S.S.); 2 Department of Biological, Chemical, and Pharmaceutical Science and Technology (STEBICEF), University of Palermo, 90123 Palermo, Italy; carla.gentile@unipa.it; 3 CREA Council for Agricultural Research and Economics—Research Centre for Cereal and Industrial Crops of Acireale, 95024 Catania, Italy; fabiola.sciacca@crea.gov.it (F.S.); nino.virzi@crea.gov.it (N.V.); massimo.palumbo@crea.gov.it (M.P.)

**Keywords:** durum wheat bread, *Portulaca oleracea* L., essential fatty acids, omega-6/omega-3 ratio, antioxidants

## Abstract

The addition of functional ingredients to breads could have effects on preventing cardiovascular diseases, cancers and inflammation. The incorporation of 0–5–10–15% of three populations of dried purslane flour on the rheological, sensorial and nutritional quality of fortified durum wheat breads were evaluated. The increase in dried purslane (up to 15%) caused an increase in the resistance to the mixture and a consequent reduction in its extensibility. The “panel test” gave a largely positive evaluation in 10% of enrichment. The fatty acids in breads resulted higher with the 5% substitution. Contrary to what has been imagined, the increase in percentage of substitution to 10 and 15% did not lead to an increase in linoleic (omega-3) and α-linolenic (omega-6) acid and probably the cause is in the cooking. The total phenols content and the antioxidant potential, evaluated by ferric reducing antioxidant potential (FRAP) and 2,2′-azinobis (3-ethylbenzothiazoline-6-sulfonic acid) (ABTS) assays of the enriched breads increased with the percentage of the dry purslane substitution. The enrichment of the durum wheat flour with 5% purslane resulted in a good compromise to obtain good rheological characteristics of loaves and breads with decreased omega-6/omega-3 ratio and good antioxidant properties.

## 1. Introduction

Functional foods can be considered those whole, enriched or enhanced foods, that provide health benefits in addition to basic nutritional functions, when consumed as part of a varied diet on a regular basis.

Recent consumer interest in nutrition and health has increased the commercial demand for functional foods [1,2]. Functional foods can also be obtained by fortifying the product with so called phytochemicals; components with high nutritional value found in nature in different types of plants. Foods fortified with phytochemicals have been associated with the prevention of at least four frequent causes of death: cancer, cardiovascular diseases, high blood pressure, and diabetes [1,2,3,4]. Interests in incorporating bioactive ingredients, plant materials, herbs or spices, rich in bio-compounds, into popular foods such as bread, have grown rapidly due to the increased consumer health awareness [5]. Bread is the staple food of the Mediterranean diet and is appreciated and eaten in countries around the world. The bread produced basically from wheat flour, is rich in carbohydrate and provides more than 50% of the total energy intake. Due its relatively low cost, availability, acceptability, and widespread consumption, bread is an excellent product in which the incorporation of functional ingredients, especially omega-3 fatty acids, is attempted [6]. In recent decades, different research teams have worked on fortifying bread with natural compounds due to the demands for healthier food [7,8]. Thus, whole grains and seeds are commonly used in the production of bread [9,10]. Moreover, a technical challenge for food technologists is the production of breads with greater volume, smoother texture and good shelf life, possessing the quality characteristics derived from the functional properties of fats.

*Portulaca oleracea* L. (purslane) is an annual herbaceous plant with reddish stems and alternate leaves from the family Portulacaceae. Purslane is distributed in many parts of the world and specifically the tropical and subtropical areas. The aerial parts of the plant are somewhat crunchy, have a slight lemony taste and are consumed as salads [11,12]. It is a well known plant in traditional medicine; its medicinal value is evident from its use as purgative, cardiac tonic, emollient, muscle relaxant, anti-inflammatory and diuretic with immune-protective properties [13,14,15].

Purslane has been described as a power food due its high nutritive and antioxidant properties and activities, mainly acting as a free radical scavenger, metal quencher and lipid peroxidation inhibitor, thanks to its phenolic constituents and several fatty acids. Purslane is abundant in ω-3 fatty acids, particularly in α-linolenic acid (0.83 mg g^−1^), for which it is considered one of the richest plant sources. Apart from α-linolenic acid, which represents nearly up to 30% of purslane oil, other essential fatty acids have also been detected in plant tissues, such as palmitoleic, palmitic, linoleic, oleic and stearic, acids, as well as trace amounts of 20:5ω-3 and 22:6ω-3, namely eicosapentaenoic acid and docosahexaenoic acid, respectively [12,16]. In a number of regions in southern Italy, bread is mainly made from durum wheat, and different varieties have been identified as suitable for both bread and pasta production [17,18]. In this study, the effect of incorporating different amounts of dried purslane flour on the technological, sensorial and nutritional quality of fortified durum wheat breads was evaluated.

## 2. Materials and Methods

### 2.1. Plant Material

Purslane germplasm was collected in three different sites of eastern Sicily: Caltagirone (Cal; 37°11′07′′ N-14°13′19′′ W), Cassibile (Cas; 36°58′33′′ N-15°12′18′′ W) and Santa Venerina (SVen; 37°40′23′′ N-15°19′26′′ W) during June 2017. In the identified sites of collection, the plant resulted widespread and naturally covered the degraded soils. The biomorfological and chemical characterization of the plant material were reported in a previous study [12]. Linolenicacid resulted as the most abundant (0.86 mg g^−1^), followed by the palmitic (0.76 mg g^−1^) and oleic acids (0.25 mg g^−1^) in dried whole plants [7]. The collected parts of the plant were blended thoroughly for homogeneity and washed with deionized water. After draining the excess water, the biomass (leaves + stalks) was dried at 40 °C for 3 days and ground into powder (about 300 micron). The final moisture content was less than 8%.

### 2.2. Bread-Making Test

Each form of bread was obtained adding to 400 g of commercial durum wheat flour (xg semolina + xg dried purslane) 8 g of sucrose, 8 g of salt, 24 g dehydrated mother yeast and x mL of distilled water, calculated according to the water absorption index (WA) by Brabender farinograph analysis. The obtained mixture was divided into two 200 g shapes and placed in rectangular aluminum pans of 23 × 11 × 5 cm. For each purslane population, different concentrations (5%, 10% and 15% w/w) of the substitution on the total weight of the flour were studied. Bread without purslane was used as the control (CTRL). The different types of produced bread are reported in Table 1. Three replicates were prepared for each bread sample. The bread loafs were square-shaped in an experimental aluminum box, in leavening conditions of 30 °C for 1 h, 75% r.h. and bakedin a Polin mod. Wind Pierre experimental oven (Verona. Italy), at 180 °C, for 18 min. The bread samples (Cal5, Cal10, Cal15, Cas5, Cas10, Cas 15, SVen5, SVen10 and SVen15, Table 1) were then subjected to the instrumental measurement for volume (Geass Volumometer, cm^3^) and the height of the loaf of breads (Vernier Caliper, cm). 

### 2.3. Rheological Characteristics

The dough-mixing properties of the control and the different mix were examined with the Brabender Farinograph (Brabender, Duisburg, West Germany) according to the constant flour weight procedure (AACC n° 54–21). Three hundred grams’ flour was mixed at the optimum water absorption and the farinograph curve was centered on the 500 BU line. According to the standard procedure, the following farinograph indices were determined: (1) water absorption of blend (WA), (2) development time of dough (DT), (3) stability of dough (S), and (4) the degree of softening of dough (DS) (Table 2). The alveographic test was used to analyze the effect of the additions on the dough rheological behavior performed by Chopin alveograph (Chopin, Villeneuve La Garenne, France) according to the standard alveographic (UNI n° 10453 method) (American Association of Cereal Chemists 2000). Each sample was analyzed in triplicate and the deformation energy W (strength) and P/L (tenacity/extensibility ratio) were calculated.

### 2.4. Sensorial Analysis of Bread

Bread samples were submitted to a panel of 10-trained tasters (five men and five women, aged between 27 and 60 years) in order to evaluate the sensory attributes. The panel group was an on-going panel with prior training. The panelists were selected based on their sensory skills (ability to accurately determine and communicate the sensory attributes as the appearance, odor, flavor and the texture of a product). The samples (CTRL and 5%, 10% and 15% of the fortified bread of all three populations) were served in dishes randomly labeled with three-digit random numbers for all panelists. The beads were sliced (1 cm thick) and were offered in distinct dishes at the same time. Water was provided for rinsing purposes. They were evaluated on crust thickness, elasticity, hardness, friability, apparent softness (force required for compressing the bread slice on a flat surface with a finger, to obtain a deformation about 50% of crust); on crumb elasticity, friability, cohesiveness, humidity, optical evaluation of the average size and homogeneity of the alveoli, cohesiveness to the crust. We asked the panelists to give a bread overall judgment for the overall taste and odor. To this end, a 10-point scale was used: 1—low sensation, 10—high sensation while for overall taste and odor and final overall judgment, 1corresponds to extremely unpleasant and 10 to extremely pleasant. The threshold of acceptability was set at 6.

### 2.5. Bread Colour Evaluation

Bread color data were collected with the use of a Minolta colorimeter CR, 400, as previously described by Melilli et al. [19]. The colorimeter was calibrated using the manufacturer’s standard white plate (L* = 96.55; a* = −0.35; b* = −0.16), where the L* value represents light–dark spectrum with a range from 0 (black) to 100 (white), the a* value represents the green–red spectrum with a range from −60 (green) +60 (red). The b* value represents the blue–yellow spectrum with a range from −60 (blue) +60 (yellow) (CIELAB color space) [20].

### 2.6. Fatty Acids Content

The fatty acids content was determined in the germplasm collected [12], in the CTRL and in the breads enriched with purslane. The fatty acid composition was studied on 0.5 g amounts of samples after the saponification of triglycerides, as described in Melilli et al. [12], after the fatty acids were transformed into corresponding methyl esters (FAMEs) and injected in a gas chromatography–mass spectrometry (GC/MS) (Thermo-Fisher) [21,22]. Using Thermo Scientific Xcalibur Data system software for Windows, the peak areas were determined and identified by the comparison of the retention times with those of a FAMEs standard mix (Supelco CRM18918 SUPELCO FAME Mix C8–C24) separated under the same chromatographic conditions. Triplicate analyses were prepared for each sample, and the analyzed FAMEs were expressed in mg g^−1^.

### 2.7. Total Phenols Content 

The total phenol content in the bread samples (TPC) was determined using the Folin–Ciocalteu method as reported by Singleton, et al. [23], with some modifications [24]. One gram of fine bread powder was extracted with 10 mL of a solution MeOH:H_2_O (80:20). After sonication for 40 min, the extracts were filtered and stored in a −20°C freezer overnight. For the determination of the TPC, 625 μL of the Folin–Ciocalteu reagent (Merck, Darmstadt, Germany, diluted 5 times) and 1.2 mL of Na_2_CO_3_ (7% w/v) solution were added to 125 μL of the samples’ extracts. The mixtures were vortexed for two minutes and incubated in the dark for 1 h. Absorbance at 760 nm was measured using a spectrophotometer (Eppendorf Bio Spectrometer^®^ basic). The TPC was expressed as mg gallic acid equivalent in 100 g of dried bread samples (mgGAE 100 g^−1^d.m.). For every sample, the protocol was repeated three times.

### 2.8. Antioxidant Activity

Bread samples with added purslane at 5 and 10% as well as the CTRL were submitted to analysis. Two aliquots of 5 g of each dried bread sample was extracted three times with 15 mL of 70% ethanol (v/v) distilled water. After a cleanup step via centrifugation (10 min at 10,000× *g*, 4 °C), and filtration through a Millex HV 0.45 μm filter (Millipore, Billerica, MA, USA), the supernatants of each extraction cycle were recovered, combined, and used for the analysis of antioxidant activity. The antioxidant properties of the extracts from bread samples were evaluated using two antioxidant assays: the ABTS (2,2′-Azino-bis(3-ethylbenzothiazoline-6-sulfonic acid) assay and the ferric reducing antioxidant power (FRAP) assay. The ABTS assay was performed according to Re et al. [25] and the FRAP assay was performed according to Benzie and Strain [26]. The calibration curve was constructed using Trolox, an hydrophilic analogue of vitamin E. The samples were analyzed at five different dilutions, within the linearity range of the assay, as described by Gentile et al. [27]. All the measurements were repeated two times and values expressed as µmol Trolox equivalent (TE) 100 g^−1^. 

### 2.9. Data Analysis

Data were submitted to the Bartlett’s test for the homogeneity of variance and then analyzed using an analysis of variance (ANOVA). Means were statistically separated on the basis of a Student–Newmann–Kewls test, when the ‘F’ test of the ANOVA for treatment was significant at least at the 0.05 probability.

## 3. Results and Discussion

### 3.1. Rheological Characteristics

The results of the rheological characteristics (Table 2) show that the origin of the purslane did not result in significant differences in the properties of the dough; on the contrary, the different percentages of substitution of wheat semolina with dried purslane induced highly significant differences both on the parameters detected by alveograph and farinograph.

It is known that gluten is composed of glutenin and gliadin subunits: the first ones give the dough toughness, while the gliadins determine its extensibility. The alveographic indices are normally related to the quality and quantity of gluten; in particular, the *p* value indicates the dough tenacity and the L value, its extensibility. The P/L configuration ratio indicates the balance between the two factors. As shown in previous works, the P/L parameter greatly affects the bread-making quality and a value of this ratio close to unity is favorable to the baking process [17].

Table 2 shows that a partial substitution (from 5 to 10%) of wheat semolina with dried purslane induced significant modifications on the alveographic parameters (P/L and W). By comparing the CTRL sample and the formulation containing 5% dried purslane, the W value decreased by about 50%; on the contrary, the P/L value tripled. In the formulation containing 10% dried purslane, the W value decreased by about 43% and the P/L value increased, indicating inextensible dough. Moreover, when the substitution level increased, up to 15%, the alveographic indices could not be determined, indicating that the high substitution level was opposed to the dough development. The farinographic parameters included the water absorption, development time and the dough stability. Table 2 shows that the value of all the farinographic indices increased with the increase in the percentages of substitution. In particular, it shows the greatest variation of water absorption among farinograph indices, which is in agreement with the observations of Wang et al. [28]. The partial replacement, from 5 to 15% wheat semolina by the dried purslane, led to an increase in water absorption (Table 2). As for the development time, only the mixture with 15% dried purslane showed a significant increase in DT value (7.50 min). Concerning S during mixing, the addition of different percentages of dried purslane caused a progressive increase in the values, in particular in the formulation containing 15% dried purslane. Significantly, S increased from 4.2 min (no addition) to 16.90 min (15%).

### 3.2. Color, Form and Organoleptic Characteristics of the Fortified Breads

The color of the food is the first parameter of quality evaluated by consumers. The objective values of CIELAB, on crust and crumb, the height and the volume of the bread samples, subjected to sensory analyses, are reported in Table 3, as the average of the percentage of substitution and of the purslane origin. The color indices were not affected by the type of purslane added both for the crust and crumb, that on average, had L* values of 41.35 (crust) and 47.87 (crumb). 

The same behavior was noticed for the visual appearance of the loaf of breads (high 5.61 cm with a volume of 263.19 cm^3^ as average). Statistical differences in color were recorded in relation to the percentage of purslane substitution for the crust and crumb; in fact, together they resulted darker, increasing from 0 (CTRL) to 15%. The height of the loaf of bread resulted improved by adding 5% purslane flours.

The addition of purslane to bread was expected to influence its structure, altering the organoleptic characteristics. For this reason, the sensory properties of the bread samples were investigated in this work were addressed (Table 4). All the samples had values ranging from 8 to 6 in the overall bread judgment until 10% substitution (Table 4), except for the Cas10 sample (5.3).

For crumb, the addition of purslane led to a darker color than for the CTRL. The thickness resulted higher than 4.0 in all the samples than in the CTRL (3.0), while the hardness sensation was higher until 10% substitution only for the Cas and Cal bread samples. SVen breads, on average of purslane fortification, gave the similar results (5.1) with respect to the CTRL (5.0). 

The elasticity of crumb resulted improved in the Cas5 and Cas10 breads, while the other samples gave values lower than the CTRL (5.0). The alveoli average size and distribution were not affected by the dry purslane addition.

In general, the addition of purslane improved the darkness, as can be expected, with an increase in the percentage of substitution. With the exception of the 15% substitution, all the bread samples had good elasticity, thickness, friability and apparent softness with very small variations between the purslane accessions. The general judgment was always close to 6 (acceptability threshold) in the bread samples (means Cas 5.8, Cal 5.8 and SVen 6.2).

### 3.3. Chemical Characterization and Antioxidant Activity

Table 5 shows the FAMEs content detected in the CTRL bread and the fortified breads. The literature data of the FAMEs of dry purslane were also reported [12]. The content of the fatty acids in breads was influenced by both the percentage of substitution and the type of purslane used to enrich the bread samples. As it is possible to observe, the content in linolenic acid, linoleic acid and oleic acid is strongly influenced by the percentage of substitution. In fact, the initial content of FAMEs detected in dry purslane influenced the final fatty acid content of the fortified breads. The CTRL bread sample showed good levels of total FA (∑fatty acids 221.2 mg 100 g^−1^). The results obtained showed a 54% increase in Cas5, 23% in SVen5 and 10% in Cas10. On the contrary, no increase in content was observed in all the other bread samples.

Regarding α-linolenic acid (ALA), 8.4 mg 100 g^−1^ was the concentration of this fatty acid in the CTRL, but its content was higher in the all samples analyzed, in particular in the Cal5 (13.2 mg 100 g^−1^) Cas5 (16.6 mg 100 g^−1^) and SVen5 (13.2 mg 100 g^−1^). The linoleic acid concentration in CTRL was 132.3 mg 100 g^−1^, and increased considerably in Cas5 (203.0 mg 100 g^−1^) and SVen5 (143.8 mg 100 g^−1^). In general, for both Cas5 and SVen5, there was an increased concentration of PUFA compared to the CTRL (140.7 mg 100 g^−1^) of 219.6 and 156.9 mg 100 g^−1^, respectively. Focusing the contents of ALA and LA in dry purslane [7], we would have expected an increase with higher purslane substitution, which instead was not observed. We hypothesized that the baking process may have altered the structure of the PUFAs. Regarding the ω6/ω3 ratio, which in the CTRL was 15.7 mg g^−1^, this decrease was observed in all the samples and was within a range of 8.6 and 11.2 mg g^−1^.

These data agree with Giaretta et al. [29], that found the addition of kinako flour and chia seed to bread resulted in a significant increase in the content of polyunsaturated fatty acid (PUFA) with a lower ω6/ω3 ratio. The effect of the baking process was mainly directed on the ω6/ω3 ratio.

The importance of the ratio of the ω6/ω3 essential FA is well examined by Simopoulos [30]. According to the author, western diets are deficient in ω3 fatty acids, and have excessive amounts of ω-6 fatty acids, promoting the pathogenesis of many diseases, including cardiovascular disease, cancer, and inflammatory and autoimmune diseases, whereas increased levels of omega-3 PUFA (a low ω-6/ω-3 ratio) exert suppressive effects. 

**Figure 1:**
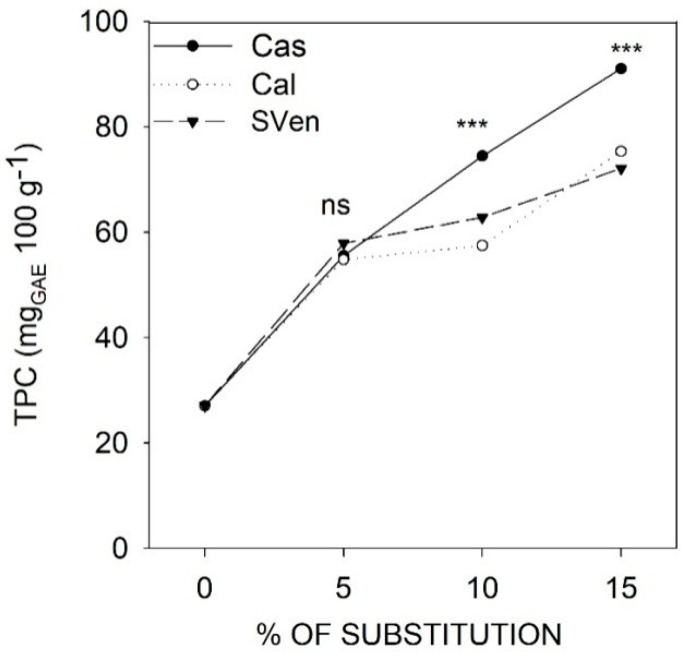
The total phenol content (TPC) of the fortified breads, obtained with durum wheat flour and purslane at three different concentrations. (ns) indicates not statistically different. (***) significant at 0.001 probability level. LSD (Least Significant Difference) (*p* < 0.05) Cas = 15.3; LSD (*p* < 0.05) Cal = 12.8; LSD (*p* < 0.05) SVen = 14.2.

On the contrary of FA, the TPC content, reported in Figure 1, increased with the percentage of dry purslane substitution. Common bread presented a TPC content of 27 mgGAE 100 g^−1^. On the average of the three types of purslane, adding 5% dry purslane the TPC content was double (56 mgGAE100 g^−1^). At 10 and 15% substitution, the effect of the dry purslane added resulted statistically different. The increase in TPC, using Cal purslane resulted 75 (Cal 10) and 91 mgGAE100 g^−1^ (Cal 15), while using Cas or SVen purslane, the increase in TPC was smaller (73 mgGAE100 g^−1^, value averaged at 15% of Cas and SVen substitution).

**Figure 2:**
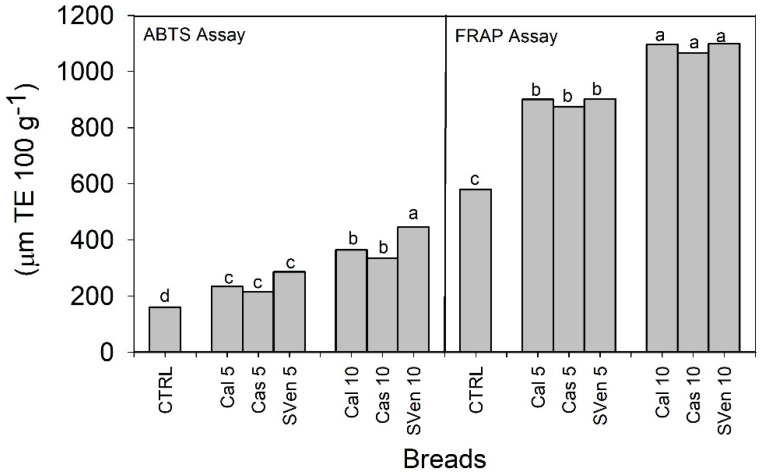
Trolox equivalent (TE) antioxidant activity (FRAP assay and ABTS assay) of the fortified breads obtained with durum wheat flour and the three populations of purslane at 5 and 10% substitution. Different letters among bars indicate significant differences at 0.05 probability level. FRAP: ferric reducing antioxidant power); ABTS 2,2′-azinobis (3-ethylbenzothiazoline-6-sulfonic acid).

Considering the results of the sensory properties of the fortified breads, the antioxidant properties, in terms of FRAP and ABTS, were analyzed until 10% purslane substitution. The FRAP (ferric reducing antioxidant power) assay was based on the reduction of ferric ion by phenols and the ABTS assay with 2,2′-azinobis(3-ethylbenzothiazoline-6-sulfonic acid) was based on free radical scavenging [16,17,18]. Results are graphed in Figure 2. An increase in antioxidant activity is one of the main aims of food supplementation. Both assays (FRAP and ABTS assays) highlighted the capacity of purslane to increase the antioxidant potential of enriched samples vs. the CTRL. We decided to focus the interest on the samples with the lower substitution, also in view of the best values of ALA content, TPC and antioxidant potential activity.

Regarding the ABTS results: breads with 5% purslane had an antioxidant activity of 244.7 µmol TE 100 g^−1^ vs. 159.9 µmol TE 100 g^−1^ in CTRL. The results of the FRAP assay had the same trend: also in this case, values did not result statistically different among the types of purslane, that on average had an antioxidant potential of 8921 vs. 580 µmol TE 100 g^−1^ in CTRL. 

## 4. Conclusions

The rheological characteristics of the wheat flour dough enriched with dried purslane provided useful information on the modifying effect of these additions on the behavior of the dough during its development, mixing and testing. However, the fundamental difference in the rheological effect of the additions was that the increase in the dose of dried purslane caused a reduction in its extensibility.

The inclusion in the wheat flour of plants rich in bioactive compounds certainly gave fortified bread good potential in relation to health benefits. Even though the increasing interest of consumers in these products, the food development and food design have to find a good compromise between the percentage of substitution of plant/herbs or spice and the sensorial properties. In our work, the enrichment of durum wheat flour by up to 5% purslane resulted in a good compromise to obtain the good rheological characteristics of loaves and breads with a decrease inomega-6/omega-3 essential FA ratio and good antioxidant properties. Considering in the Sicilian and Mediterranean tradition, common bread is made mainly using durum wheat, the addition of only 5% dry purslane could be a useful strategy to increase the bioactive compounds with potential health benefitsin bread. Trials concerning the shelf life of the products are ongoing.

## Figures and Tables

**Table 1:** Bread samples.

Origin	Code	Percentage of Substitution		
		5%	10%	15%
Cassibile	Cas	Cas5	Cas10	Cas15
Caltagirone	Cal	Cal5	Cal10	Cal15
S. Venerina	SVen	SVen5	SVen10	SVen15

**Table 2:** Alveographic and farinographic indices of the doughs enriched with purslane averaged for concentrations and populations. Different letters indicate differences at *p* < 0.05 (according to the Duncan test).

Populations	Alveograph Analysis		Farinograph Analysis		
	W (strength) (10^−4^ J)	P/L (tenacity/extensibility ratio)	Water Absorption %	Development Time (min)	Stability (min)
			Average of concentrations		
Cas	86 a	7.79 a	64.92 c	3.47 a	9.35 a
Cal	86 a	7.91 a	65.50 a	3.45 a	9.20 a
SVen	88 a	8.50 a	65.05 ab	5.45 a	8.97 a
Means	86.78	8.07	65.16	4.13	9.18
			Average of populations		
0	135 a	3.58 b	62.90 d	2.00 b	4.20 d
5	67 b	10.15 a	64.40 c	2.57 b	6.13 c
10	58 b	10.48 a	65.70 b	4.43 b	9.47 b
15	-	-	67.63 a	7.50 a	16.90 a
Means	86.78	8.07	65.16	4.13	9.18

**Table 3:** L*, a*, b* in the breads enriched with purslane on the average of concentrations and populations. Different letters indicate differences at *p* < 0.05.

Populations	Crust L*	Crust a*	Crust b*	Crumb L*	Crumb a*	Crumb b*	Height cm	Volume cm^3^
Average of concentrations								
Cal	42.01 a	9.96 a	19.59 a	49.00 a	1.47 b	16.34 a	5.53 a	265 a
Cas	41.18 a	9.97 a	18.69 a	47.57 b	2.10 a	15.83 a	5.59 a	260 a
SVen	40.88 a	10.38 a	19.55 a	47.04 b	2.04 a	16.19 a	5.71 a	263 a
Means	41.35	10.10	19.28	47.87	1.87	16.12	5.61	263
Average of populations								
0	46.82 a	16.69 a	28.09 a	69.26 a	−0.28 d	22.58 a	5.67 b	336 a
5	39.74 c	9.98 b	19.02 b	44.80 b	2.04 c	15.35 b	5.98 a	259 b
10	37.06 d	8.21 c	14.55 d	40.30 c	2.71 b	14.03 c	5.48 b	240 bc
15	41.79 b	5.53 d	14.88 c	37.11 d	3.01 a	12.52 d	5.32 b	217 c
Means	41.35	10.10	19.14	47.87	1.87	16.12	5.61	263

**Table 4:** Sensory attributes of the crust and crumb of the fortified breads with purslane and their overall judgment. The threshold of acceptability for the bread’s overall judgment is 6.

Sensory Attributes	Crust										Means					
		Cas			Cal			SVen								
	CTRL	5	10	15	5	10	15	5	10	15	Cas	Cal	SVen	5%	10%	15%
Thickness ^a^	3.0	4.0	3.0	4.0	4.0	4.0	5.0	4.3	5.0	4.0	4.0	4.3	4.4	4.1	4.0	4.3
Elasticity ^a^	5.5	5.5	6.0	6.0	6.0	8.0	6.0	4.0	4.0	4.0	5.5	6.7	4.0	5.2	6.0	5.3
Hardness ^a^	5.0	6.0	6.0	4.0	6.0	6.0	4.0	5.3	4.0	6.0	6.0	5.3	5.1	5.8	5.3	4.7
Friability ^a^	4.0	3.0	2.0	4.0	4.0	4.0	4.0	2.0	2.0	6.0	3.0	4.0	3.3	3.0	2.7	4.7
Apparent softness ^a^	3.0	3.0	2.0	4.0	4.0	4.0	3.0	4.0	2.0	4.0	3.0	3.7	3.3	3.7	2.7	3.7
Crumb																
Elasticity ^a^	5.0	6.0	6.0	4.0	4.0	6.0	4.0	3.0	4.0	6.0	6.0	4.7	4.3	4.3	5.3	4.7
Apparent softness ^a^	3.3	4.0	4.0	4.0	4.0	4.0	4.0	4.0	4.0	4.0	4.0	4.0	4.0	4.0	4.0	4.0
Friability ^a^	5.0	5.0	6.0	4.0	4.0	6.0	6.0	3.0	5.0	6.0	5.0	5.3	4.7	4.0	5.7	5.3
Cohesiveness ^a^	5.0	6.0	6.0	4.0	4.0	4.0	4.0	4.0	4.0	4.0	6.0	4.0	4.0	4.7	4.7	4.0
Humidity ^a^	3.8	5.0	4.0	4.0	4.0	4.0	4.0	3.0	4.0	6.0	5.0	4.0	4.3	4.0	4.0	4.7
Average size of the alveoli ^a^	3.5	4.5	2.0	3.0	3.3	2.0	4.0	3.5	2.0	3.0	4.5	3.1	2.8	3.8	2.0	3.3
Homogeneity of the alveoli ^a^	7.0	7.0	7.0	7.0	7.0	7.0	7.0	7.0	7.0	7.0	7.0	7.0	7.0	7.0	7.0	7.0
Cohesiveness to the crust ^a^	7.5	7.0	6.0	6.0	6.0	6.0	6.0	6.0	6.0	6.0	7.0	6.0	6.0	6.3	6.0	6.0
Bread overall judgment ^b^	8.0	8.0	5.3	4.0	7.3	6.0	4.0	8.0	6.7	4.0	5.8	5.8	6.2	7.8	6.0	4.0

^a^ 1—good feeling and 10—bad feeling; ^b^ 1—extremely unpleasant, 10—extremely pleasant. CTRL—bread without purslane.

**Table 5:** Fatty acid methyl esters (FAME) content (mg 100 g^−1^) in the fortified breads. Different letters indicate statistical differences at *p* < 0.05 among percentage of substitution into the same purslane population.

	Fortified Bread Sample										Dry Purslane *		
	CTRL	Cal5	Cal10	Cal15	Cas5	Cas10	Cas15	SVen5	SVen10	SVen15	Cal	Cas	SVen
Palmitic a.	42.0	41.0 a	35.0 b	32.4 c	65.1 a	47.4 b	29.3 c	48.6 a	38.5 b	35.1 b	39.0	42.0	35.0
Oleic a.	38.5	35.5 a	30.6 b	26.2 c	56.9 a	40.0 b	24.1 c	41.4 a	32.9 b	28.2 b	20.0	21.0	10.0
LA	132.3	119.3 a	104.3 b	90.3 c	203.0 a	141.1 b	83.6 c	143.8 a	115.1 b	93.7 c	88.0	78.0	51.0
ALA	8.4	13.2 a	9.3b	10.5 b	16.6 a	13.0 a	7.7 b	13.1 a	9.5 b	9.9 b	84.0	89.0	67.0
∑fatty acids	221.2	209.0 a	179.2 b	159.5 c	341.5 a	241.5 b	144.6 c	246.8 a	196.0 b	166.9 c	172	167	118
PUFA	140.7	132.5 a	113.6 ab	100 b	219.6 a	154.1 b	91.3 c	156.9 a	124.6 b	103.6 c	231	230	163
PUFA/SFA	3.3	3.2 a	3.2 a	3.1 a	3.4 a	3.2 a	3.1 a	3.2 a	3.2 a	2.9 b	4.41	3.98	3.37
ω-6/ω-3 ratio	15.7	9.0 b	11.2 a	8.6 b	12.2 a	10.9 b	10.9 b	10.9 b	12.1 a	9.4 b	1.05	0.88	0.76

* [12]; (LA: linoleic acid; ALA: α-linolenic acid; PUFA: polyunsaturated fatty acid; SFA: saturated fatty acid).
